# Paths to Autonomous Motivation and Well-being: Understanding the Contribution of Basic Psychological Needs Satisfaction in Health Professions Students

**DOI:** 10.1007/s40670-024-02106-9

**Published:** 2024-07-05

**Authors:** Yuanyuan Zhu, Diana Dolmans, S. Eleonore Köhler, Rashmi A. Kusurkar, Latifa Abidi, Hans Savelberg

**Affiliations:** 1 Department of Educational Development and Research, School of Health Professions Education, Faculty of Health, Medicine and Life Sciences, Maastricht University, Universiteitssingel 60, 6229 ER Maastricht, The Netherlands; 2 School of Health Professions Education, Faculty of Health, Medicine and Life Sciences, Maastricht University, Universiteitssingel 50, 6229 ER Maastricht, The Netherlands; 3 Research in Education, Amsterdam UMC Location Vrije Universiteit Amsterdam, De Boelelaan 1118, 1081 HZ Amsterdam, The Netherlands; 4 LEARN! Research Institute for Learning and Education, Faculty of Psychology and Education, Amsterdam UMC Location Vrije Universiteit Amsterdam, Amsterdam, The Netherlands; 5 Amsterdam Public Health, Quality of Care, Amsterdam, The Netherlands; 6 Department of Health Promotion, Faculty of Health, Medicine and Life Sciences, Maastricht University, Peter Debyeplein 1, 6229 HA Maastricht, The Netherlands; 7 School of Health Professions Education, Faculty of Health, Medicine and Life Sciences, Maastricht University, Universiteitssingel 50, 6229 ER Maastricht, The Netherlands

**Keywords:** Autonomous motivation, Self-Determination Theory, Basic psychological needs, Well-being, Structural Equation Modelling, Health professions education

## Abstract

**Background:**

Undergraduate students enrolled in Health Professions (HP) programs may experience challenges related to motivation and well-being. According to Self-Determination Theory, learning environments that support the three basic psychological needs (needs for autonomy, relatedness, competence) foster students’ autonomous motivation and well-being. Little is known about the associations between basic psychological needs satisfaction, autonomous motivation, and well-being in the HP domain and how they relate to one another in an integrative model. This study assesses the associations of the path “basic psychological needs satisfaction-autonomous motivation-well-being” within HP.

**Methods:**

We invited first-year students in the field of HP (*N* = 850) to fill out an electronic survey, measuring the satisfaction of each basic psychological need, autonomous motivation, and well-being and performed structural equation modelling to examine the paths between these variables.

**Results:**

In total, 202 students completed the survey (response rate 23.8%). Our model had an acceptable model fit: CFI = 0.924, TLI = 0.916, RMSEA = 0.052, SRMR = 0.057, chi-square test of model fit = 688.678 (*p* < .001). Autonomy satisfaction was directly and positively associated with autonomous motivation. The satisfaction of relatedness and competence was directly and positively associated with well-being, and each of them explained approximately the same degree of strength in well-being. Autonomous motivation did not have a direct effect on well-being.

**Conclusion:**

When students perceived their programs as autonomy supportive, they might develop higher autonomous motivation. Fostering students’ relatedness and competence might enhance students’ well-being. Teachers and curriculum designers can consider developing learning environments that support students’ autonomy, relatedness, and competence.

## Background

Undergraduate students in Health Professions (HP) programs face the challenge of decreasing levels of autonomous motivation and well-being. Autonomous motivation, according to the Self-Determination Theory (SDT), involves behaviors with a full sense of volition and choice [1]. Individuals who act for autonomous reasons are more likely to engage in and maintain a behavior intrinsically, without relying on external reinforcement or contingencies [2]. Autonomous motivation is composed of different types of regulations. Autonomously motivated students perform a task because they accept the values of the task (identified regulation), integrate the values with the self (integrated regulation) [3], or genuinely find the task interesting (intrinsic motivation) [4]. In addition to autonomous motivation, SDT distinguishes controlled motivation and amotivation. Controlled motivation relies more on external reasons such as guilt avoidance and the desire to please others (introjected regulation) and win rewards (external regulation). Amotivation indicates the absence of any intention to perform [5].

Autonomous motivation is important for students because it is positively related to higher academic performance [6, 7] and improved well-being [1]. However, students’ autonomous motivation has been reported to significantly drop at the end of the first year compared with the motivation at the beginning [8] and in the last year compared with the first 4 years [9]. A decline has been observed in the positive indicators of students’ well-being (e.g., life satisfaction [10]) and an increase in negative factors (e.g., anxiety [8]) in the last year of their study compared to the first years. Understanding and improving students’ autonomous motivation and well-being remain a challenge in HP education.

According to SDT, supporting the three basic psychological needs (autonomy, relatedness, and competence) in learning environments facilitates students’ autonomous motivation [11] and well-being [12]. The need for autonomy refers to the sense of initiative and ownership [3]. The need for relatedness implies connectedness and a sense of belonging. The need for competence pertains to feeling effective in actions one pursues and performs [13]. When students’ basic psychological needs are supported, they are more likely to move toward autonomous motivational states and to be more autonomously engaged in their studies [14], because their behavior is congruent with their own interests, values, and goals [15]. Students have a tendency to internalize the values and behaviors within such settings, as they are encouraged to engage in behaviors that hold personal significance, foster group connection, and empower them to take action [4].

In addition to the positive effect of the three needs on autonomous motivation, SDT theorizes that needs fulfillment is directly associated with improved well-being [16]. SDT research has typically used subjective well-being as one of several indicators of well-being [16]. Therefore, our study also explores subjective well-being, defined as “people’s overall evaluations of their lives and emotional experiences” [17, 18]. It entails two components: affective (i.e., positive and negative emotions) and cognitive (i.e., life satisfaction) [18, 19]. SDT posits that satisfaction of the basic psychological needs fosters subjective well-being [16]. The three needs define the requirements and delineate the nutriments for psychological health, which is linked to the two components of subjective well-being [16].

Motivation has also been found to be associated with subjective well-being [20, 21]. Autonomous motivation is associated with different aspects of subjective well-being: positively with life satisfaction and positive emotions but negatively with negative emotions [5]. Given its strong connection to the satisfaction of basic psychological needs, it is a logical inference to suggest that autonomous motivation plays a mediating role in the relationship between basic psychological needs satisfaction and well-being [22]. Previous studies have tested the mediating role of autonomous motivation when studying the contribution of basic psychological needs satisfaction on well-being across multiple domains and cultural contexts, supporting the mediating effect of autonomous motivation [15, 22]. In the current study, we focus on autonomous motivation because of its positive relationship with basic psychological needs and well-being and a lack of research on this positive path of basic psychological needs-motivation-outcome [23].

After reviewing the literature, we identified several gaps. Firstly, while some studies examined the associations between these variables, the findings were not fully consistent. On the one hand, basic psychological needs satisfaction was found to be associated with autonomous motivation [22] and well-being across domains [24–26]; on the other hand, not all three needs were found to be related to autonomous motivation or well-being. Associations between satisfaction of autonomy and relatedness with autonomous motivation were validated, but not between competence satisfaction and autonomous motivation [15, 27]. Satisfaction of relatedness and competence was associated with well-being while autonomy was not [15]. In another study, which also used self-reported measures, the satisfaction of autonomy and competence was associated with indices of well-being whereas relatedness satisfaction was not [28]. Although the SDT literature indicates a mediating role of autonomous motivation, studies found only partial mediation of basic psychological needs satisfaction on well-being via autonomous motivation [15, 22]. Secondly, there is a scarcity of research that assesses the integrative model of associations between basic psychological needs satisfaction, autonomous motivation, and well-being or indicators of well-being within the realm of HP programs, in contrast to the ample studies in general education [29, 30]. Among the limited number of studies within HP programs, the majority of them studied the associations between two of the three variables [31–34] or with a mediating variable such as affect [35] and resilience [36]. There is a need of quantitative studies in HP programs to understand the integrative pathway of “needs-motivation-consequences” using methods like structural equation modelling or path analysis [23]. Previous studies mostly used correlation or regression analysis [21, 31, 37] and ignored relatedness satisfaction and its associations with other variables [23].

Understanding the associations between basic psychological needs satisfaction, autonomous motivation, and well-being in the HP context is important to enhance education since there is a dearth of policies that reflect the constructs of basic psychological needs in the domain of HP education such as in medical education [36]. It is particularly important for HP educators to understand the associations and accordingly create learning environments that support students’ autonomous motivation and well-being, which are essential to prepare HP students for their future roles in healthcare [38].

## The Present Study

**Fig. 1:**
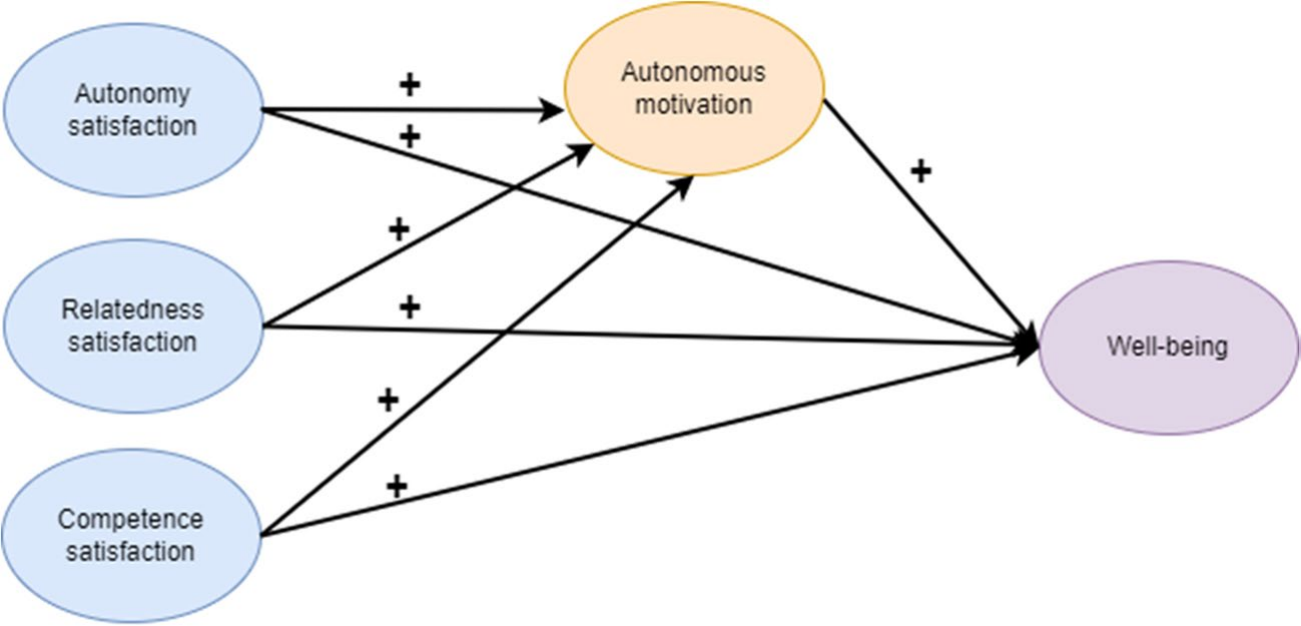
Hypothesized model: The satisfaction of the three basic psychological needs stimulates autonomous motivation, and autonomous motivation is independently and positively associated with well-being. The paths connecting these variables are represented by arrows

The present study aims to reveal a comprehensive underlying mechanism of SDT by assessing the associations between the basic psychological needs satisfaction, autonomous motivation, and well-being in the HP domain. Figure 1 displays the hypothesized model and associations between the variables. It may provide insights into how each need contributes to autonomous motivation and well-being and what role autonomous motivation plays in this path, adding an integrative model to the SDT literature in the HP field. We anticipate this study to offer a valuable framework for enhancing and evaluating current study programs in a manner that fosters students’ autonomous motivation and promotes their well-being.

### Research Questions

1. How is the satisfaction of basic psychological needs (autonomy, relatedness, competence) associated with autonomous motivation?
2. How is autonomous motivation associated with well-being?
3. How is the satisfaction of basic psychological needs (autonomy, relatedness, competence) directly associated with well-being and indirectly through autonomous motivation?

## Methods

### Participants

At the end of the academic year, we invited first-year students from three undergraduate programs (*N* ≈ 350, 250, 250 for A, B, C, respectively) at Maastricht University in the Netherlands to fill out an anonymous electronic survey. The survey design ensured respondents could not skip questions except for age. All responses were recorded, including the incomplete surveys. Students received an email about the survey through the university online platform and paper-based flyers. They received a reminder email 1 month after the first announcement through the same online platform. Students in all three programs have two semesters in an academic year, and each semester consists of two periods of 8 weeks and one period of 4 weeks. Students in all three programs have the same study load. Programs A and B have 12 h of classes and 28 h of individual study per week. Students in program C have 14–18 h of classes and 25 h of individual study per week. All three programs adopt a range of assessment methods as written or oral exams, peer feedback, and oral presentations. Programs A and B are typical majors within the HP field, and program C is an interdisciplinary program that allows students to choose courses from diverse fields, including courses in the HP field such as neurosciences and biomedical engineering. The survey was open for 8 weeks from June to September 2021 (including a summer break). During this period, students experienced blended education, consisting of online lectures and a mix of online and in-person tutorials. Participants gave informed consent in the survey before participation.

### Measures

The survey included demographic questions (age and gender) and the following three scales (Appendix 1) to measure basic psychological needs satisfaction, autonomous motivation, and well-being. The Basic Psychological Needs Satisfaction and Frustration Scale measures perceived satisfaction and frustration of basic psychological needs. Its measurement has been cross-culturally validated and has shown good reliability and construct validity [39]. The full scale consists of 24 items that measure the satisfaction and frustration of autonomy, relatedness, and competence. Students rated statements on a 5-point Likert scale ranging from 1 (completely disagree) to 5 (completely agree) (Table 2). For the current study, we explored only the satisfaction of the three needs, using 12 items of the scale (four for each need) since we focused on the positive relationships of needs satisfaction with motivation and well-being.

The full Academic Motivation Scale [40] contains 28 items and measures the quality of students’ academic motivation based on the SDT [32, 41]. Students were asked to rate items on a 7-point Likert scale ranging from 1 (does not correspond at all) to 7 (corresponds exactly) how closely a list of reasons reflected their motivation to study. It examines autonomous and controlled motivation with their sub-regulations (i.e., intrinsic, identified, introjected, external), as well as amotivation. For the purposes of this study, we included only the 16 items related to autonomous motivation (intrinsic and identified regulations), which was calculated as the mean score of the intrinsic and identified regulations.

The WHO (Five) Well-Being Questionnaire [42] is a brief measure with non-invasive questions into respondents’ subjective well-being, and its measurement has shown high validity [43]. It has been translated into over 30 languages and used in research projects all over the world [44]. Individuals were asked to indicate for each of the five statements how they felt, using a 6-point Likert scale ranging from 0 (at no time) to 5 (all of the time). We slightly modified the wording of the stem in the scales measuring basic psychological needs satisfaction and autonomous motivation to align with participants’ experiences in their study programs over the past academic year. For the scale measuring well-being, participants completed the survey based on their experiences over the past 2 months, which aimed to capture a recent yet broader impression of students’ experiences.

### Analysis

We used IBM SPSS Statistics 26.0 to check for normal distribution of data. We calculated Pearson’s correlations to examine the relationships between the variables. We checked the internal consistency of all scales using Cronbach’s alpha coefficients. To ensure the reliability and validity of our findings, we excluded surveys that were less than 50% completed. One-way analysis of variance (ANOVA) followed by Bonferroni’s post hoc comparisons tests was applied to understand whether students’ gender (Table 1) was related to their autonomous motivation and well-being. The mean difference was considered significant at the 0.05 level.

**Table 1:** Participants’ program, age, and gender

	Program A	Program B	Program C	Total
*N*	103 (51%)	61 (30%)	38 (19%)	202
Age	19.7	19.7	19.7	Mean = 19.7
Gender				
Female	76 (74%)	48 (78%)	31 (81%)	155 (77%)
Male	26 (25%)	12 (20%)	6 (16%)	44 (22%)
Other	1 (1%)	1 (2%)	1 (3%)	3 (1%)

The mean difference was considered significant at the 0.05 level

Structural equation modelling using MPlus 8.1 was conducted to test the hypothetical model. Confirmatory factor analysis was performed (the measurement model). We used bootstrapping with 1000 iterations to test the mediation effect of autonomous motivation between autonomy, relatedness, and competence satisfaction, with well-being. We referred the results to fit indices including the Comparative Fit Index (CFI), Tucker–Lewis Index (TLI), root mean square error of approximation (RMSEA), and standardized root mean square residual (SRMR). CFI and TLI > 0.90, and RMSEA and SRMR < 0.08 indicated an acceptable model fit [45]. We calculated the *p* value of chi-squared differences between different models to decide the final model using the output from MPlus and Excel.

## Results

### Demographics and Descriptives of the Variables

After excluding the dataset that was less than 50% completed, a total of 202 responses remained, which were all fully completed (students in programs A = 103, B = 61, and C = 38; Table 1). Five respondents did not provide their age. We marked these missing age values as discrete missing values and calculated the mean age using the available data. The mean ages of students were the same for all three programs (19.7 years). There were 155 female and 44 male participants, and three participants chose the category “Other.” ANOVA showed that there were no significant effects of gender on students’ autonomous motivation [*F* (2, 199) = 0.309, *p* = 0.735, partial eta squared = 0.003] or well-being [*F* (2, 199) = 3.024, *p* = 0.051, partial eta squared = 0.029]. Although well-being had a *p* value close to the chosen significance threshold (*p* = 0.051), the smallest *p* value in the follow-up post hoc tests was 0.058 (between male and female participants). This further supported the conclusion that well-being did not differ between genders. Students’ mean scores varied across the five variables (Table 2).

**Table 2:** Scales, Cronbach’s *α*, mean, and standard deviation (SD) of participants’ scores in the eight variables

Measure	Scale	Variable	Cronbach’s *α*	Min	Max	Mean	SD	Skewness	Kurtosis
Basic psychological needs satisfaction and frustration scale (4 items each need)	1 Not true 5 Completely true	Autonomy	0.583	1.3	4.5	3.5	0.61	− 0.759	0.612
		Relatedness	0.841	1.0	5.0	3.6	0.75	− 0.379	0.512
		Competence	0.872	1.5	5.0	3.6	0.73	− 0.205	− 0.478
Academic motivation scale (16 items)	1 Does not correspond at all 7 Correspond exactly	Autonomous motivation	0.908	1.6	7.0	5.2	0.88	− 0.560	0.743
WHO-5 well-being Index (5 items)	0 At no time 5 All the time	Well-being	0.831	0.6	4.4	2.6	0.87	− 0.382	− 0.548

The standard errors of skewness and kurtosis are 0.171 and 0.341, respectively (for all variables)

### Correlations Between the Variables

Pearson’s correlation analysis revealed moderate positive correlations [46] between competence satisfaction and well-being (*r* = 0.512), relatedness satisfaction and well-being (*r* = 0.489), autonomy satisfaction and autonomous motivation (*r* = 0.458), autonomy satisfaction and competence satisfaction (*r* = 0.436), and weak positive correlations between all remaining variables (Table 3).

**Table 3:** Correlations between satisfaction of the three basic psychological needs, autonomous motivation, and well-being

Variables	Autonomy	Relatedness	Competence	Autonomous motivation	Well-being
Autonomy	-				
Relatedness	0.348**	-			
Competence	0.436**	0.367**	-		
Autonomous motivation	0.458**	0.297**	0.328**	-	
Well-being	0.380**	0.489**	0.512**	0.316**	-

**Correlation was significant at the 0.01 level (2-tailed)

### Measurement Model

The measurement model was adjusted based on factor loadings and modification indices. All Cronbach’s alpha coefficients were greater than 0.70 except autonomy satisfaction (0.583) due to the poor inter-relatedness of the third item (“I felt my choices expressed who I really am”). It had a low factor loading (0.229) and high residual variance (0.947). We therefore removed the third item measuring autonomy satisfaction. Modification indices from MPlus output suggested that some items on the WHO-5 Well-Being Index were related to each other, which was not fully captured by the well-being factor in our initial model. Specifically, they were between the first and second items (“I felt cheerful and in good spirits” and “I felt calm and relaxed”) and between the second and fourth items (“I felt calm and relaxed” and “I woke up feeling fresh and rested”). We examined the theoretical implication of these five items that while being derived from different scales, all indicated positive feelings within the global dimension of well-being [44]. Therefore, we added covariances between these two sets of items, improving the model’s accuracy in representing the data. Essentially, the adjustment acknowledges that the measured effects of these two items often occurred together beyond what our initial well-being factor represented. After the adjustment, all factor loadings ranged between 0.446 and 0.967 (*p* < 0.001) with acceptable fit indices: CFI = 0.924, TLI = 0.916, RMSEA = 0.052, and SRMR = 0.057, chi-square test of model fit = 688.569 (*p* < 0.001). We removed the non-significant path with the lowest path coefficient, specifically the one between relatedness satisfaction and autonomous motivation, resulting in model 1: CFI = 0.925, TLI = 0.917, RMSEA = 0.051, SRMR = 0.057, chi-square test of model fit = 688.678 (*p* < 0.001). The non-significant difference (*p* = 0.810) between the chi-squared values of the measurement model and model 1 indicated there was no need to further remove other paths (see Appendix 2 for comparison between the measurement model and model 1). The unadjusted model (measurement model) was used as the final model because it preserved the information of the path between relatedness satisfaction and autonomous motivation and did not differ significantly from model 1.

### Structural Model

**Fig. 2:**
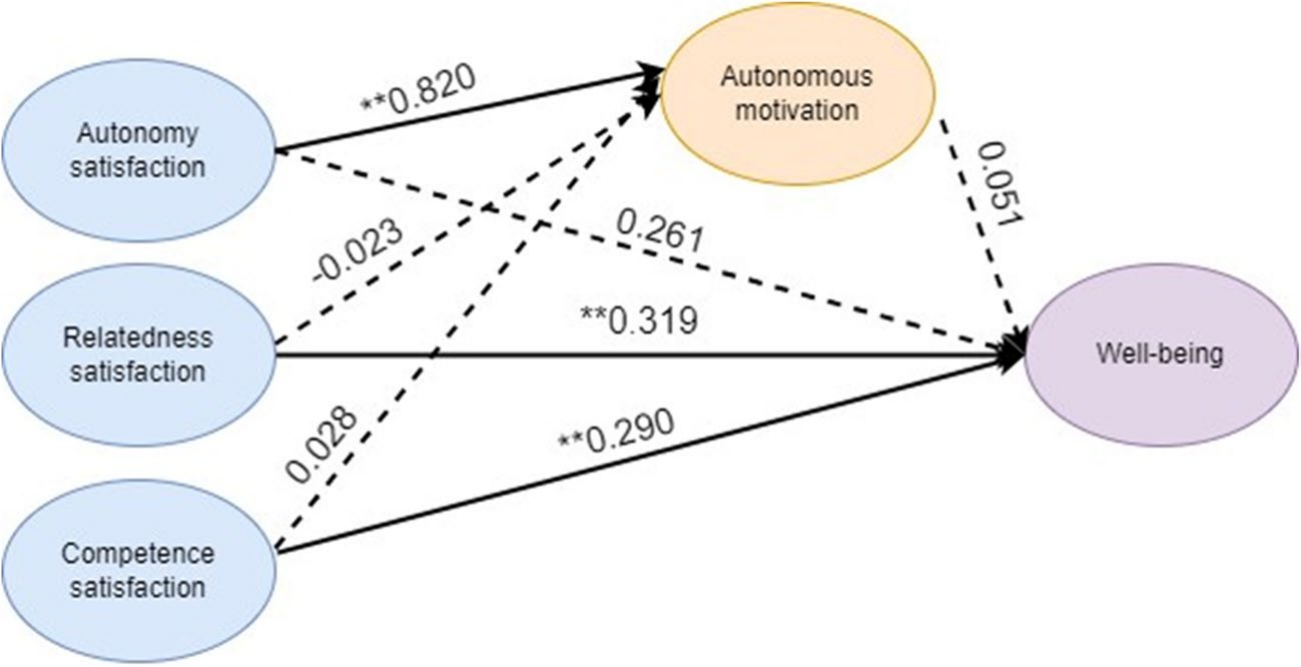
Final structural model of relations between satisfaction of autonomy, relatedness, competence with autonomous motivation, and well-being. Autonomy satisfaction was positively and directly linked with autonomous motivation. Satisfaction of relatedness and competence was positively and directly associated with well-being, while autonomous motivation was not associated with well-being. The significant and non-significant paths connecting these variables are represented by line arrows and dotted arrows, respectively

Autonomy satisfaction was directly and positively associated with autonomous motivation (*β* = 0.820, *p* < 0.001) (Fig. 2). Neither relatedness satisfaction (*β* =  − 0.023, *p* = 0.861) nor competence satisfaction (*β* = 0.028, *p* = 0.832) was associated with autonomous motivation. Satisfaction of relatedness (*β* = 0.319, *p* = 0.001) and competence (*β* = 0.290, *p* = 0.007) was directly and positively associated with well-being. Autonomy satisfaction (*β* = 0.261, *p* = 0.385) and autonomous motivation (*β* = 0.051, *p* = 0.839) were not directly associated with well-being. Neither autonomy (*β* = 0.061, *p* = 0.886), relatedness (*β* =  − 0.002, *p* = 0.988) nor competence (*β* = 0.002, *p* = 0.975) satisfaction had indirect effects on well-being via autonomous motivation.

## Discussion

Our findings highlight the important role of autonomy satisfaction in fostering autonomous motivation. We observed direct and positive associations between the satisfaction of relatedness and competence with well-being. The results underscored the significance of satisfying all three needs to cultivate students’ autonomous motivation and well-being. This study contributes to our understanding of the intricate relationships among basic psychological needs satisfaction, autonomous motivation, and well-being by delving into the integrative model of these variables.

Building upon our exploration of the integrative model and literature, we elaborate the key findings. Autonomy was significantly associated with autonomous motivation. This result aligns with the SDT proposition that autonomy satisfaction is linked with autonomous motivation [47] since autonomy and autonomous motivation represent a similar concept of choice. Previous studies similarly reported that autonomy accounts for the most substantial portion of the variance in autonomous motivation, in contrast to the other two needs [27], or that autonomy satisfaction is the only need associated with autonomous motivation [33].

We did not find significant associations between satisfaction of relatedness and competence with autonomous motivation. One possible explanation is that data points of autonomous motivation were negatively skewed compared with other variables, which might reduce the statistical significance of its associations with other variables. Most data points on autonomous motivation ranged between 4 and 7 on a 7-point Likert scale, and only two data points scored less than 3. The relatively concentrated distribution on the right side of the scale indicated that our participants had a rather high autonomous motivation. However, this narrow range of distribution made it challenging to detect associations with other variables. Even though we adopted the robust method of maximum likelihood to estimate the parameters in the model, skewness might still have affected the strength of the associations with the satisfaction of relatedness and competence.

Satisfaction of relatedness and competence was related to well-being, which aligns with some of the previous studies in which only one or two of the needs were related to well-being [15, 48, 49]. Our results supported SDT in that basic psychological needs exert direct and universal effects on well-being [16]. Intriguingly, one study [26], which also used structural equation modelling, reported that autonomy was the need that most strongly related to indicators of well-being, while our results did not find a significant path between autonomy satisfaction and well-being. We found that the paths between both relatedness and competence to well-being had approximately the same degree of strength, implying the equal importance of the satisfaction of both needs for well-being. Our result resonates with a previous study, which explored the relation of balanced needs satisfaction with well-being [50]. People who experienced balanced needs satisfaction tended to report higher well-being than those with the same sum score but greater variability in needs satisfaction. Results from both our study and that previous study aligned with SDT’s proposition that all basic psychological needs are essential for well-being and should not vary much in their importance for different people (i.e., all needs are important). What varies is the extent to which people’s needs are satisfied (i.e., different groups experience different degrees of needs satisfaction) [50].

There was no significant association between autonomous motivation and well-being, nor a mediation effect from basic psychological needs to well-being via autonomous motivation. These results contradict not only the SDT literature that autonomous motivation is associated with greater well-being [1], but also previous studies that explored the mediation effect of autonomous motivation, whether adopting three needs separately [15] or as a single score [22]. One explanation to this non-significant path was the distribution of autonomous motivation scores as discussed before; i.e., participants had a rather high autonomous motivation. A second reason could be that potential mediators weakened the association between autonomous motivation and well-being. Given the context of the study, we speculate that the COVID-19 pandemic had reduced the explanatory power of autonomous motivation on well-being. The survey investigated participants’ experience in their past academic year during which students experienced distance learning, which had been found to influence students’ autonomous motivation [29, 51] and well-being [52, 53]. Previous studies using the same motivation scale on HP students reported higher means of autonomous motivation, such as 5.5 [54] and 5.3 [6], both greater than the mean of 5.2 in our study. Similarly, well-being scores in other studies were higher; for example, a study evaluating the psychometric properties of the same well-being scale among university students reported a mean of 3.5 [55], greater than the mean of 2.6 in our study. We suspect that these two possible reasons weakened the explanatory power of autonomous motivation on well-being.

### Implications for Teachers and Curriculum Developers

The findings of this study offer valuable insights for teachers and curriculum developers into options to enhance students’ autonomous motivation and well-being. Autonomy satisfaction was positively related to autonomous motivation, and the satisfaction of relatedness and competence had positive associations with well-being. Thus, it is pivotal to support all three needs in learning environments. Teachers and curriculum designers are recommended to adopt learning activities that support students’ basic psychological needs. They can offer students opportunities for personal choices such as elective clerkships and research projects [56]. While offering students the freedom, a balance between letting students take the lead and providing support, for example by providing case study protocols [56] and structured teaching sessions [57], also has to be maintained. We encourage teachers and curriculum designers to incorporate authentic learning situations into the curriculum, which help students develop a sense of self and confidence as professionals [58, 59]. These situations need to be relevant to students’ work reality, e.g., by introducing a real patient [59]. Teachers and curriculum designers can consider adopting a student-centered small-group teaching approach such as problem-based learning. This approach allows students to work collaboratively and creates a satisfactory feeling of mastery [13].

### Limitations

This study had a few limitations. Firstly, the participants exclusively consisted of students enrolled at the same university in the Netherlands. While this avoided variations on the university level, the narrow participant pool might limit the generalizability of the findings. The sample size was sufficient based on the rule-of-thumb for structural equation modelling with a minimum sample size of 200 [60]. However, the low response rate might have compromised the generalizability and induced the risk of desirability bias; i.e., mostly students with a relatively high autonomous motivation might have responded. Secondly, although the model fit supported the associations between variables indicated in the SDT, it is important to note that causal inferences could not be drawn due to the cross-sectional design. Certain terms in this article may inadvertently have conveyed a sense of causality, as we strived to describe relationships within the framework of the SDT, which inherently implied such causations. Thirdly, we did not test the model in different subgroups such as gender and study program because the size of some subgroups did not suffice for structural equation modelling.

### Future Research Directions

Firstly, future researchers are recommended to enhance the generalizability of a similar study by using diverse participant sampling such as participants with various study programs and cross-cultural backgrounds. This will help to assess whether the universal principles of the SDT also apply in different domains and with different participants. Although sample size was sufficient, future research might consider model-specific patterns of association between parameters and sample size [60]. Secondly, it might be beneficial to adopt alternative designs and methods such as a longitudinal and experimental design or a mixed methods design. A longitudinal and experimental design could contribute to a more comprehensive exploration of these associations between the variables over time, and mixed methods might provide insights that explain the survey data. Thirdly, after establishing the model, future researchers could apply the model to subgroups of participants (e.g., gender, study program, ethnic background), providing insights of how the paths might vary depending on the characteristics of such subgroups.

## Conclusions

This study adds to the SDT literature on the positive path of basic psychological needs satisfaction-autonomous motivation-well-being and provides insights to what extent HP students’ experiences in learning environments reflect this path. Autonomy satisfaction was directly and positively associated with autonomous motivation and the two variables correlated strongly. The satisfaction of relatedness and competence was directly and positively associated with well-being. In other words, students’ well-being tended to be related with feeling related and competent. This study adds to an integrative understanding of students’ autonomous motivation and well-being. Teachers and curriculum developers are encouraged to nurture students’ autonomy, relatedness, and competence to help students become autonomously driven with enhanced well-being.

## Figures and Tables

**Fig. 3:**
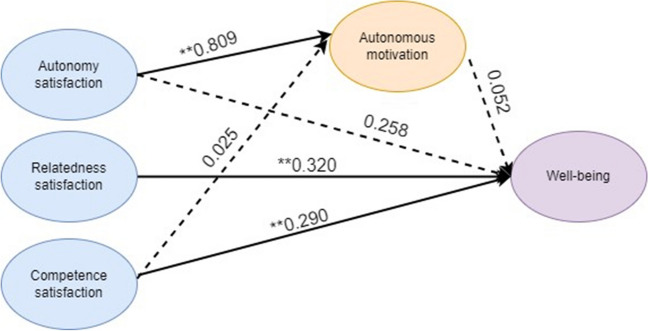
Model 1 (adjusted model) without the path between relatedness satisfaction and autonomous motivation

## Data Availability

The datasets used and/or analyzed during the current study are available from the corresponding author upon reasonable request.

## Appendix 1

Three scales in the survey.

**Table:**

Basic Psychological Needs Satisfaction and Frustration Scale	
Study Program Experience Below, we ask for your experiences during the first year of your study program. Please choose a value from 1 to 5 to indicate the degree to which the statement is true for you In my study program:	1-Not true 2 3 4 5-Completely true
1. I felt a sense of choice and freedom in the things I undertook	Autonomy
2. I felt that the people I care about also care about me	Relatedness
3. I felt confident that I could do things well	Competence
4. I felt that my decisions reflect what I really wanted	Autonomy
5. I felt connected with people who care for me, and for whom I care	Relatedness
6. I felt capable at what I did	Competence
7. I felt my choices expressed who I really am	Autonomy
8. I felt close and connected with other people who are important to me	Relatedness
9. I felt competent to achieve my goals	Competence
10. I felt I have been doing what really interests me	Autonomy
11. I experienced a warm feeling with the people I spent time with	Relatedness
12. I felt I could successfully complete difficult tasks	Competence
Academic Motivation Scale	
Reasons Enrolled in the Program Please indicate to what extent each of the following items presently corresponds to one of the reasons why you enrolled in your study program I enrolled into my study program because:	1-Does not correspond at all 2 3 4 5 6 7-Correspond exactly
1. I experience pleasure and satisfaction while learning new things	Intrinsic motivation—to know
2. I think that this study program will help me better prepare for the career I have chosen	Identified
3. For the intense feelings I experience when I am communicating my own ideas to others	Intrinsic motivation—to experience stimulation
4. For the pleasure I experience while surpassing myself in my studies	Intrinsic motivation—toward accomplishment
5. For the pleasure I experience when I discover new things never seen before in this field	Intrinsic motivation—to know
6. Because eventually it will enable me to enter the job market in a field that I like	Identified
7. For the pleasure that I experience when I read interesting authors in this field	Intrinsic motivation—to experience stimulation
8. For the pleasure that I experience while I am surpassing myself in one of my personal accomplishments	Intrinsic motivation—toward accomplishment
9. For the pleasure that I experience in broadening my knowledge about subjects which appeal to me	Intrinsic motivation—to know
10. Because this will help me make a better choice regarding my career orientation	Identified
11. For the pleasure that I experience when I feel completely absorbed by what certain authors in this field have written	Intrinsic motivation—to experience stimulation
12. For the satisfaction I feel when I am in the process of accomplishing difficult academic activities	Intrinsic motivation—toward accomplishment
13. This study program allows me to continue to learn about many things that interest me	Intrinsic motivation—to know
14. I believe that a few additional years of education in this study program will improve my competence as a worker	Identified
15. For the “high” feeling that I experience while reading about various interesting subjects in this field	Intrinsic motivation—to experience stimulation
16. This study program allows me to experience a personal satisfaction in my quest for excellence in my studies	Intrinsic motivation—toward accomplishment
WHO-5 Well-being Index	
Feelings and Well-being Please indicate for each of the five statements which is closest to how you have been feeling over the last 2 months	0-At no time 1-Some of the time 2-Less than half of the time 3-More than half of the time 4-Most of the time 5-All the time
1. I felt cheerful and in good spirits	Well-being
2. I felt calm and relaxed	
3. I have felt active and vigorous	
4. I woke up feeling fresh and rested	
5. My daily life was filled with things that interest me	

## Appendix 2

Process of model adjustment.

We started with removing the non-significant path with smallest loading based on the results of the measurement model (Fig. 2), i.e., the path between relatedness satisfaction and autonomous motivation (model 1, Fig. 3).

**Table:**

Index differences between the measurement model and model 1									
	CFI	TLI	RMSEA	SRMR	*R*^2^	S.E. for *R*^2^	*X*^2^	df	*p* value for *X*^2^
Measurement model	0.924	0.916	0.052	0.057	Autonomous motivation = 0.680	Autonomous motivation = 0.087	688.569	448	0.810
					Well-being = 0.544	Well-being = 0.082			
Model 1	0.925	0.917	0.051	0.057	Autonomous motivation = 0.676	Autonomous motivation = 0.087	688.627	449	
					Well-being = 0.544	Well-being = 0.079			

## Abbreviations

*HP*: Health Professions
*SDT*: Self-Determination Theory
